# SEM evaluation of pulp reaction to different pulp capping materials in dog’s teeth

**Published:** 2007-01-20

**Authors:** Saeed Asgary, Masoud Parirokh, Mohammad Jafar Eghbal, Jamileh Ghoddusi

**Affiliations:** 1 *Department of Endodontics, Iranian Center for Endodontic Research, Dental School, Shaheed Beheshti University of Medical Sciences, Tehran, Iran*; 2 *Department of Endodontics, Dental School, Kerman University of Medical Sciences, Kerman, Iran*; 3 *Department of Endodontics, Iran Center for Dental Research, Dental School, Shaheed Beheshti University of Medical Sciences, Tehran, Iran*; 4 *Department of Endodontic Department, Dental School, Mashad University of Medical Sciences, Mashad, Iran*

**Keywords:** Calcium enriched mixture, Calcium hydroxide, MTA, Pulp capping agent, SEM

## Abstract

**Introduction:** This investigation evaluates the effects of mineral trioxide aggregate (MTA), calcium hydroxide (CH) and calcium enriched mixture (CEM) as pulp capping materials on dental pulp tissues.

**Materials and Methods:** The experimental procedures were performed on eighteen intact dog canine teeth. The pulps were exposed. Cavities were randomly filled with CEM, MTA, or CH followed by glass ionomer filling. After 2 months, animals were sacrificed, each tooth was sectioned into halves, and the interface between each capping material and pulp tissue was evaluated by scanning electron microscope (SEM) in profile view of the specimens.

**Results:** Dentinal bridge formation as the most characteristic reaction was resulted from SEM observation in all examined groups. Odontoblast-like cells were formed and create dens collagen network, which was calcified gradually by deposition of calcosphirit structures to form newly dentinal bridge.

**Conclusion:** Based on the results of this *in vivo* study, it was concluded that these test materials are able to produce calcified tissue in underlying pulp in the case of being used as a pulp capping agent. Additionally, it appears that CEM has the potential to be used as a direct pulp capping material during vital pulp therapy.

## Introduction

Direct pulp capping (DPC) is defined as usage of a suitable dental material over an exposed pulp to protect it from additional injury and also initiates the formation of irritation dentin at the site of injury. This technique permits preservation of pulp tissue to continue its functions (1). Pulp capping is mainly indicated for reversible pulp tissue injury of developing or mature teeth. Inducing the dentinogenic potential of pulpal undifferentiated mesanchymal cells as the ultimate goal of DPC material application is widely accepted (2). Various factors including pulp status, root formation stage, periodontal condition, age, time elapsed between pulp exposures and protection, pulp protecting materials, the nature and size of exposure and bacterial microleakage can influence the ultimate result of pulp preservation (3). Although a wide variety of various dental materials have been used for DPC procedures, but the most pertinent include different formulations of calcium hydroxide (CH), dentin-bonding materials, and mineral trioxide aggregate (MTA).

Hess reported a technique for pulpotomy using CH in 1929 (4). CH has widespread use for protecting the exposed dental pulp. As it promoted reparative dentine bridge formation and maintained pulp vitality, this material was thought to be more biologically acceptable (5). The antibacterial effect of CH on infected pulp tissue is of its considerable importance (6). Although the beneficial effect of CH is mainly attributed to the initial low-grade irritation of the traumatized underlying pulp tissue_ due to OH^-^ release _ (7) but the exact mechanism by which CH forms a dentin bridge has not been explained yet (8).

Franz *et al. *(9) studied the ultrastructure of the dentinal bridge formed under CH. They reported the presents of necrotic tissue in the layer adjacent to CH. Mentioned disadvantages for this material were its degradation over the time, tunnel defects in dentinal bridges and its poor sealing properties (10). At the present time, DPC with CH remains an unpredictable method for treatment of mature teeth.

MTA, which was introduced in 1993 by Torabinejad at Loma Linda University, has been tested since 1996 as potential DPC material of choice (11-12). Pitt Ford* et al*. have reported that the MTA, which was placed in mechanically exposed pulps of mandibular incisors in monkeys, stimulates pulp healing with minimal inflammatory reactions and dentinal bridge formation (11). MTA stimulates significantly more hard-tissue formation, and results in less inflammation demonstrated remarkable success compared with CH (12-14). The authors have documented, in a SEM study, that the most characteristic reaction of pulp cells was the intimate connection of cell processes and secretion extra-cellular fibers with the crystals of MTA. They also found the progressive mineralization of dentinal bridge formation from the periphery towards the central area (15). Although MTA has superior biocompatibility in comparison with CH, it has a delayed setting time (16), poor handling characteristics (17), while it is an expensive material.

The advantages and disadvantages of different commercial types of MTA have been documented (18-19). These preliminary data were used in a project on production of a new dental material which benefits from superior biocompatibility of MTA and also appropriate setting time (less than 1 hour), handling characteristics, chemical properties, and reasonable price. This novel endodontic cement (CEM) was formulated using different calcium compounds (20). The results of our other studies (unpublished data) revealed that the mixed CEM comprises water-soluble calcium and phosphate, and immediately forms hydroxyapatite during and after setting time. This cement form an effective seal against entrance of microorganisms, has antibacterial effect, resistant to washout, and also it is able to set in an aqueous environment.

The aim of present *in vivo* study was to evaluate the dentinogenic activity of CEM on mature dog teeth and compare it with MTA and CH by using the scanning electron microscope.

## Materials and Methods

Eighteen intact canine teeth with healthy periodontium in five healthy 18- 24 months old beagle dogs were used. All experimental procedures were carried out according to protocols approved by the Ethics Committee of Dental Research Center of the Shaheed Beheshti Medical University (M/8512). Under general anesthesia gained with intramuscular injection of 20 mg/kg Ketamine hydrochloride (Alfasan, Woerden, The Nederlands) and 1 mg/kg Xylazine (Bayer, Munich, Germany), dog teeth were polished and washed with 0.2% chlorhexidine gluconate. Intraoral anesthesia for mandibular teeth was achieved by anesthetic block injection of 1.8 ml of mepivacaine 3% (ESPE, Dental AG, Seefeld, Germany), and in maxilla by infiltration injection of 1.8 ml mepivacaine 3%. The teeth were then isolated by rubber dam. Using a No. 1 round bur (Dentsply, Tulsa, Ok, USA) in an air turbine with sterile saline spray, Class V cavities were prepared at labial surfaces of the teeth, and pulp exposures (approximately 1 mm in diameter) were obtained. Bleeding was controlled by irrigation with sterile saline solution and the cavities were dried with sterile cotton pellets before placing test materials. The cavities were randomly divided in to three test groups.

In group 1, the exposed pulps were capped with ProRoot MTA (Tooth-colored formula, Dentsply, Tulsa, Ok, USA) which was mixed according to the manufacturer’s directions to provide a grainy, sandy mixture. Using CH (LD Caulk, Milford, DE, USA) which was mixed according to the manufacturer’s directions, the pulp exposures were capped in group 2. In group 3, the calcium enriched mixture (CEM) was mixed with powder and liquid in a 3: 1 ratio to provide a putty mixture and capped the pulp exposures. A light pressure was applied with a wet cotton pellet in groups 1 and 3, resulting in an acceptable adaptation of material with the pulp tissue and cavity walls. Excess water was removed with sterile cotton pellets. The remainders of the cavities in all groups were immediately restored with Fuji II glass ionomer (GC International Corp, Tokyo, Japan) permanent filling material. In order to minimize pain and discomfort, a nonsteroidal anti-inflammatory drug (Ibuprofen, Rose Daro, Iran) was prescribed immediately following operation.The controls were selected from untreated, two intact canines on which no pulp capping were performed.

After 2 months, the animals were sacrificed by an overdose of Pentothal, and vital perfusion fixation was performed with Karnovsky solution. Then, the teeth and their surrounding tissues were removed as block sections. A crack was created on each sample by a vice. In the next step, the teeth were placed in 2.5% gluteraldehyde solution for 14 days. Following complete fixation of the samples, each tooth was carefully notched (mark) in a buccolingual direction by a diamond disc to obtain a cross-crack of filled cavity and probably underlying dentinal bridge. After washing the tooth halves with normal saline, the samples were then processed for scanning electron microscopic (SEM) analysis using critical point drying technique and then put in an oven and were allowed to dry at 50°C for 24 h.

All the samples were gold-coated using a Dynavac CS300 (Dynavac, Sydney, Australia) coating unit. A Cambridge Instruments Stereo-scan 360 (Cambridge Instruments, Cambridge, UK) SEM was used for imaging and observing basic morphology of dental pulp reaction and dentinal bridge formation in cross-sectional mode. SEM operating conditions were used as follows: 20-kV accelerating voltage, 6-mm working distance and 71-pA probe current.

## Results

The DPC procedures were well-tolerated by the experimental animals. The postoperative period was uneventful, confirming the absence of any apparent adverse effect caused by the experimental protocol.

**Figure 1:**
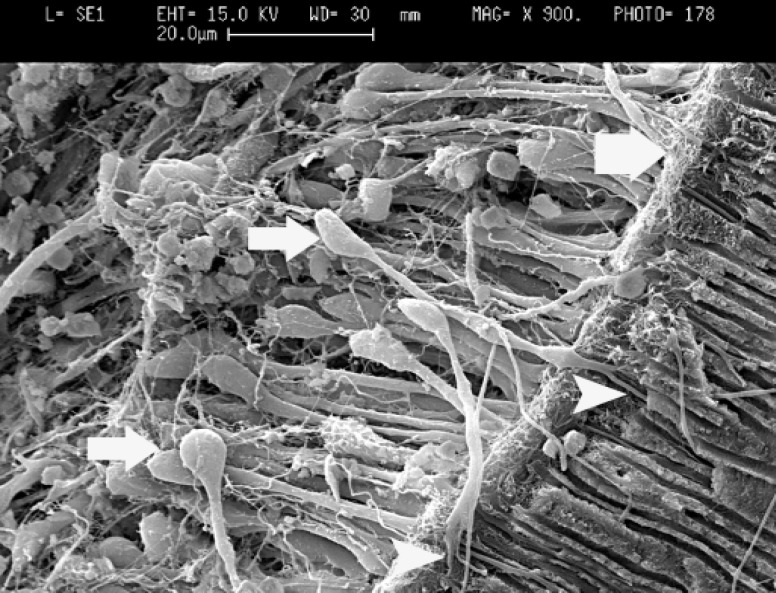
Scanning electron micrograph of dentin and pulp border of coronal pulp in control group (bold arrow: predentin layer consist of collagen matrix, normal arrows: odontoblast cell bodies in multilayer and intercellular spaces contain a fine network of reticular fibers, arrow heads: branched odontoblastic processes when entered to dentinal tubules, x900).

In the control group, the pulps were healthy and showed normal components. Odontoblast cells appear as large, closely aligned, multilayered, sweet potato- shaped cells. They averaged 3-5μm in width and 6-10μm in length. A thin layer of unmineralized dentin (predentin) was present and odontoblastic processes passed this layer and entered dentinal tubules (Figure 1).

According to statistical analysis, there was no significant difference between test groups.

**Figure 2:**
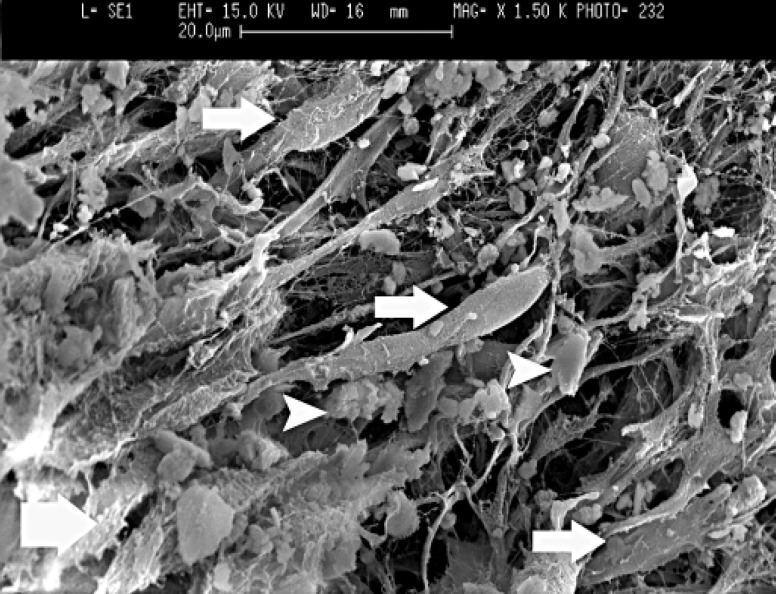
Scanning electron micrograph of dentin and pulp border in MTA pulp capped tooth (bold arrow: predentin layer consist of collagen matrix, normal arrows: odontoblast-like cell bodies longer than normal ones, arrow heads: hydroxyappatite crystals in the intercellular space, x1500).

All samples in group 1 showed vital pulp. Complete dentin bridge formation were found in all cases of direct pulp capping with MTA. Profile view of the complete dentin barrier showed that the bridges consisted of three different aspects. The outer aspect was composed of MTA in direct contact with newly formed hard tissue. In the middle portion, a dentin like bridge with irregular dentinal tubules was identified. The pulpal or inner aspects exhibited predentin layer which was wider than normal condition and it was fast mineralized. The vital pulp consists of normal elements. Young odontoblast like cells differentiated and elaborated collagen matrix and predentin layer as shown in Figure 2. In this figure, the amorphous crystals of hydroxyapatite are shown in the odontoblastic intercellular spaces as a sign of rapid mineralization process.

In group 2, vital pulp was seen in all the samples. Profile view of the barrier tissue showed that the bridges consisted of three different aspects. The outer aspect was composed of CH in direct contact with newly formed tissue. In the middle portion, a dentin like bridge in some areas was identified. The inner aspects exhibited predentin layer which was wider than normal condition, and the mineralization process was not fast.

**Figure 3:**
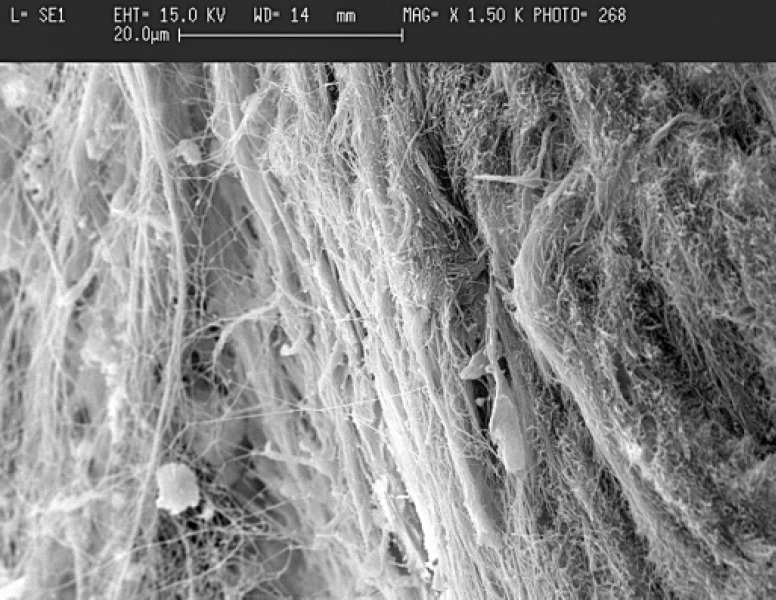
Scanning electron micrograph (SEM) of collagenous fibers of predentin of CH pulp capping (x1500).

The predentin layer made of coarse collagen bundles which were prepared for calcification (Figure 3). The pulp consists of normal elements. Odontoblast-like cells were absent.

**Figure 4:**
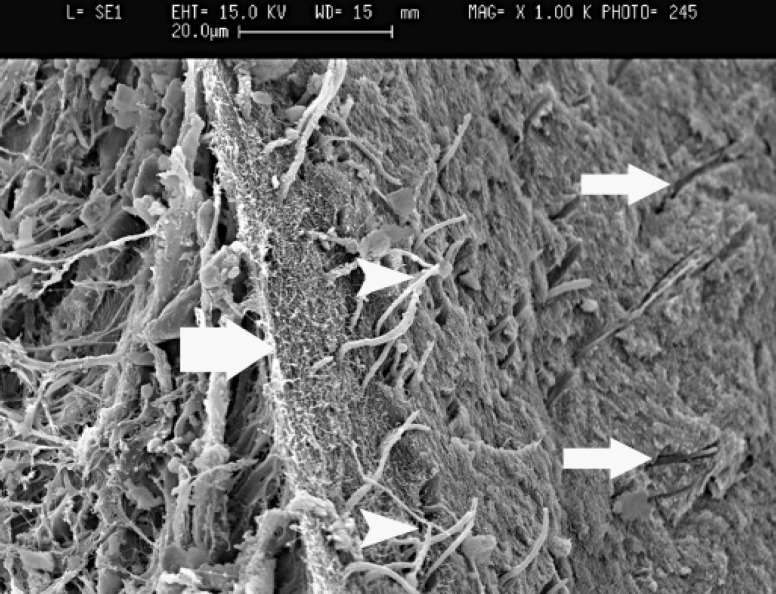
SEM of dentin and pulp (left) border in CEM pulp capped tooth. Dentinal bridge was broken obliquely and odontoblastic process exit from broken part and are seen naked (bold arrow: predentin layer consist of collagen matrix, normal arrows: cross section of dentinal tubules, arrow heads: odontoblastic processes when entered to dentinal tubules, x1000)

All samples in group 3 showed vital pulp. Complete dentinal bridge formation was found in all cases of direct pulp capping with CEM. Profile view of the complete dentin barrier showed that the bridges consisted of three different aspects. The outer aspect was composed of CEM in direct contact with newly formed hard tissue. In the middle portion, a dentin-like bridge with irregular dentinal tubules was identified. The pulpal or inner aspects exhibited predentin layer which was similar to normal condition. The vital pulp consisted of normal elements. Young odontoblast like cells were differentiated and they elaborated collagen matrix and predentin layer as shown in Figure 4. The tubular nature of dentinal bridge is shown in this figure.

## Discussion

Mature dental pulp cells possess the ability of differentiation into odontoblast-like cell lineage forming tubular dentine in the absence of normal developmental conditions, i.e. dental epithelium and basement membrane (21). Although DPC procedures are taught in general practice as a temporary treatment for carious and mechanically exposed teeth, some researchers have suggested that vital pulp therapies can be permanent (13, 22). It has been well recognized that dentinal bridge formation can be observed in exposed pulps without any exogenous application (23-24). The superficial zone of extracellular matrix at the wound surface of the repairing connective tissues is physiologically followed in exposed dental pulp by osteodentin or fibrodentin hard tissue deposition. A layer of odontoblast-like cells, producing reparative dentine, is finally formed as a sign of normalized pulp function. In morphological terms, these cells are recognized as the elongated pulpal cells with increased cytoplasm/nucleus ratio and polarized nuclei, which form tubular matrix in a polar predentin like pattern (21).

Because the pulp has enough vital tissue, it was advocated that DPC procedures could be performed successfully on an asymptomatic carious exposure (22). A clinical study showed that asymptomatic carious exposures could survive for an average of 12 yr after pulp capping (25). One of the newest DPC materials that have appeared in 1996 is MTA. The properties of MTA are well established. It has a good sealing ability, little cytotoxicity, antibacterial effect, a pH of 12.5, and biocompatibility, and also it is not affected by tissue fluid or blood contamination, (16, 26).

In the present study the pulp capped teeth with MTA showed vitality, complete dentinal bridge formation, differentiated odontoblast-like cells, collagen matrix elaboration, and predentin layer creation. Various studies have presented excellent results for MTA as DPC material (12,13,27,28). The initial effect of MTA on the surface of mechanically exposed pulp is the formation of a superficial layer of crystalline structures over the pulpal surface of the capping material. Columnar cells undergoing nuclear and cytoplasmic polarization and showing a well developed cytoplasmic organization are further arranged along the crystalline structures. Initiation of dentinogenesis can be identified only in histomorphological studies by the palisade appearance of elongated and polarized odontoblast like cell layer able to secrete tubular matrix in a polar predentin-like pattern (27). The favorable results obtained in this research on MTA are similar to the findings of previous DPC studies.

CH was used in this study, because it was shown that healing process following DPC of human teeth with CH is successful. It was reported that healing process initially consists of proliferation and migration of cells under the wound surface and elaboration of new collagen along the superficial necrotic zone or the pulpal surface of capping agent. The necrotic zone and the new collagen layer attract mineral salts, becoming calcified matrices. Then a layer of Odontoblast-like cells is formed in association with the fibrodentin, and reparative dentine is secreted (29).

In this study, the observed results of CH were as well as MTA and CEM groups. All cases showed an incomplete fibrodentin bridge and the predentin layer made of coarse collagen bundles which were not calcified in some areas. Many data from capping studies suggest that initiation of reparative dentine formation might not be attributed to any specific dentinogenic effect of CH, although its effect in controlling the infection and stimulating the wound healing process might not be excluded. It was previously reported that CH dressing of hard-setting Dycal does not provide a long-term protection against microleakage, as were dissolved within 1–2 years, and tunnel defects in the majority of the dentin bridges which allow pulp inflammation, infection or necrosis (30). This could explain the unfavorable results of the pulp capping with CH in other studies MTA is commonly used for various clinical applications, but the material is expensive causes it's restrict usage. The development of new materials that are biocompatible, bactericidal, inductive of hard tissue formation, and have similar or better sealing properties could solve this problem. Regarding favorable results of CEM we suggest this cement as one of these materials. On a comparative basis, CEM resulted in similar response to pulp capping with MTA and better than CH. Complete dentinal bridge with more thickness than CH was formed by CEM and the presence of Odontoblast-like cells was the major finding**.** This new cement contains calcium compounds such as tricalcium phosphate (TCP), calcium sulfate, calcium silicate, calcium hydroxide, calcium oxide and others to improve the biocompatibility, chemical and physical characteristics. Good biocompatibility of CEM is hypothesized to be due to its chemical properties. Different calcium compounds in CEM provide high calcium and phosphorous concentration within the mixture. These elements can produce hydroxyapatite which is a natural product of dental pulp cells (31).

However, it is unclear which components leach from CEM, but the cement contains CH and calcium oxide (which produces CH through the hydration). CH, if leached out in sufficient quantities due to a direct effect on the microvasculature resulting in less plasma outflow which, in turn, favors a calcific response in the capped tissue (32). Holland *et al.* observed granulations nearest to the opening of dentin tubes filled with MTA and implanted in an animal study. They reported that these structures are similar to the calcite crystals observed with CH (33). According to Seux *et al.* calcite crystals attract fibronectin, which is responsible for cellular adhesion and differen-tiation (34). Therefore, we hypothesized that CEM, MTA and CH have a similar mechanism for hard tissue formation. In addition, some compounds such as calcium sulfate and calcium silicate may cause a slight expansion of CEM through continuous hydration after initial setting of the material and further crystalline maturation. This may be indirectly responsible for CEM biocompatibility *via* good sealing ability.

Thus, the property of CEM may be due to its tissue reactions in similarity with MTA and CH but, in addition, providing an excellent tight seal (20).

It should be emphasized that this experiment was carried out in ideal conditions and intentionally exposed pulps of healthy teeth in animals have exclusively been studied. Careful consideration is essential for correlating these favorable results to clinical situation.

## Conclusion

In conclusion, according to the results of this *in vivo* study in dogs, CEM and MTA are effective pulp capping materials, able to stimulate hard tissue bridge formation and superior to CH cement for pulp capping procedures. Also, CEM appears to be an alternative to MTA according to their biological effects are identical.
